# Catastrophic costs incurred by tuberculosis affected households from Thailand’s first national tuberculosis patient cost survey

**DOI:** 10.1038/s41598-024-56594-1

**Published:** 2024-05-16

**Authors:** Sitaporn Youngkong, Phalin Kamolwat, Phichet Wongrot, Montarat Thavorncharoensap, Usa Chaikledkaew, Sriprapa Nateniyom, Petchawan Pungrassami, Naiyana Praditsitthikorn, Surakameth Mahasirimongkol, Jiraphun Jittikoon, Nobuyuki Nishikiori, Ines Garcia Baena, Takuya Yamanaka

**Affiliations:** 1 Mahidol University Health Technology Assessment (MUHTA) Graduate Program, Mahidol University, Bangkok, Thailand; 2 Social and Administrative Pharmacy Division, Department of Pharmacy, Faculty of Pharmacy, Mahidol University, Bangkok, Thailand; 3 Division of Tuberculosis, Department of Disease Control, Ministry of Public Health, Bangkok, Thailand; 4 Faculty of Nursing, Mahidol University, Nakhon Pathom, Thailand; 5 Department of Disease Control, Ministry of Public Health, Nonthaburi, Thailand; 6 Department of Medical Sciences, Ministry of Public Health, Nonthaburi, Thailand; 7 Department of Biochemistry, Faculty of Pharmacy, Mahidol University, Bangkok, Thailand; 8 World Health Organization Global Tuberculosis Programme, Geneva, Switzerland; 9 Department of Global Health and Development, London School of Hygiene and Tropical Medicine, London, UK; 10 School of Tropical Medicine and Global Health, Nagasaki University, Nagasaki, Japan

**Keywords:** Tuberculosis, Catastrophic total cost, Thailand, Tuberculosis, Health care economics

## Abstract

Tuberculosis (TB) causes an economic impact on the patients and their households. Although Thailand has expanded the national health benefit package for TB treatment, there was no data on out-of-pocket payments and income losses due to TB from patients and their household perspectives. This national TB patient cost survey was conducted to examine the TB-related economic burden, and assess the proportion of TB patients and their households facing catastrophic total costs because of TB disease. A cross-sectional TB patient cost survey was employed following WHO methods. Structured interviews with a paper-based questionnaire were conducted from October 2019 to July 2021. Both direct and indirect costs incurred from the patient and their household perspective were valued in 2021 and estimated throughout pre- and post-TB diagnosis episodes. We assessed the proportion of TB-affected households facing costs > 20% of household expenditure due to TB. We analyzed 1400 patients including 1382 TB (first-line treatment) and 18 drug-resistant TB patients (DR-TB). The mean total costs per TB episode for all study participants were 903 USD (95% confident interval; CI 771–1034 USD). Of these, total direct non-medical costs were the highest costs (mean, 402 USD, and 95%CI 334–470 USD) incurred per TB-affected household followed by total indirect costs (mean, 393 USD, and 95%CI 315–472 USD) and total direct medical costs (mean, 107 USD, and 95%CI 81–133 USD, respectively. The proportion of TB-affected households facing catastrophic costs was 29.5% (95%CI 25.1–34.0%) for TB (first-line), 61.1% (95%CI 29.6–88.1%) for DR-TB and 29.9% (95%CI 25.6–34.4%) overall. This first national survey highlighted the economic burden on TB-affected households. Travel, food/nutritional supplementation, and indirect costs contribute to a high proportion of catastrophic total costs. These suggest the need to enhance financial and social protection mechanisms to mitigate the financial burden of TB-affected households.

## Introduction

Tuberculosis (TB) causes a significant economic impact on the patients and their households^1,2^. Although most high TB-burden countries have offered diagnosis and treatment free of charge, patients and their households still incur substantial cost including the direct medical cost (during pre-treatment phase), direct non-medical cost (i.e., transportation, accommodation, and food), as well as indirect costs from job loss and productivity loss. Therefore, TB-affected household are still facing the risk of catastrophic costs, defined as the total costs related to TB management exceeding 20% of annual household income or expenditures^3^, leading to poor treatment access, adherence, and worsening health outcome^1,2,4^. Hence, to achieve the End TB Strategy introduced by the Sustainable Development Goals (SDGs)^5^, one of the World Health Organization (WHO)’s strategies^6^ was to eliminate the catastrophic costs among TB-affected households by 2020. According to the WHO’s global monitoring of the End TB indicators reports^7,8^, which covered the findings from the national TB patient cost survey data of the 27 countries, one in two patients (48%, 95%CI 36–67%) faces catastrophic costs. Recent modelling that produced estimates for countries that had not yet been able to complete survey^9^ shows that estimated proportions of TB-affected households experiencing catastrophic total costs were 54.9% (47.0–63.2%) overall. According to the recent meta-analysis^10^, the pooled proportion of patients faced catastrophic costs (95% Confident Interval) from the existing 29 studies was 43% (34–51%) while the main predictors of the catastrophic costs included country, drug sensitivity, and Human immune-deficiency virus (HIV) co-infection.

Thailand, an upper-middle-income country, has high TB-burden with an incidence (new TB cases per year) of 105,000 (79,000–134,000) in 2020^8^. At present, almost all necessary diagnostic and TB treatments have been covered by public health insurance schemes. As of 2019, there was no data on economic burden due to TB from patients and their household perspectives. To achieve the goal of zero catastrophic costs due to TB as one of the three targets of the WHO End TB Strategy, the current situation must be investigated. This paper is the first study aiming to estimate the prevalence of catastrophic costs due to TB from the patient and their household perspective. Factors affecting catastrophic costs were also explored. The findings could provide important evidences to guide the development of policies/strategies to protect TB patients from risk of financial crisis, hence, improving the treatment outcomes leading to the achievement of end TB target.

## Methods

### Study design

The national cross-sectional survey design and methodology were in line with WHO recommendations in their handbook for TB patient cost surveys^11^. The cost components included direct medical costs (i.e., out-of-pocket spent on diagnostic tests, medication, outpatient and inpatient care, and doctor fees), direct non-medical costs (i.e., out-of-pocket spent on transportation, food, and accommodation), and indirect costs (i.e., productivity loss due to TB) based on hourly wage computed individually from reported.

### Sample size and sampling method

We calculated the sample size based on an estimated proportion of households experiencing catastrophic costs (p) at 50%, a design effect (D.E.) of 2.0 and 4% precision level (e) with the following standard formula^12^.

$$N = D.E. \times \frac{{1.96^{2} n\left( {1 - p} \right)p}}{{e^{2} \left( {n - 1} \right) + 1.96^{2} \left( {1 - p} \right)p}}$$

where n is the total number of TB notifications registered in 2017^13^ was as 67,971; and 15% adjustment of data incompleteness, the required sample size was 1400. A stratified multi-stage cluster sampling was used to sample TB patients for the interview to ensure balance in the economic status and healthcare services accessibility of each locality that can be nationally representation in this case. Firstly, the health facilities with TB clinics were stratified into 2 groups (i.e., low- and high-poverty area) according to the poverty level (i.e., the proportion of number of individuals with income below the per capita poverty thresholds to the total number of individuals of each province compared to the national poverty proportion of 7.87^14^). Then, health facilities in each poverty level were further stratified into secondary and tertiary level, resulting in 4 stratums. The total of 40 clusters were, then, randomly selected from the 4 stratums. The number of clusters for each stratum were calculated using proportional to size approach. For each cluster, 35 patients were recruited. These resulted in 420 patients recruited from 12 clusters of tertiary hospitals in low-poverty incidence areas, 280 patients recruited from 8 clusters of tertiary hospitals in high-poverty incidence areas, 315 patients recruited from 9 clusters of secondary hospitals in low-poverty incidence area, and 385 patients recruited from 11 clusters of secondary hospitals in high-poverty incidence area). The patients were eligible if they (1) were registered for TB treatment enrolled in the National Tuberculosis Control Programs (NTPs) from October 2019 to July 2021 at sampled facility, and (2) were on treatment for a minimum of 14 days either in intensive or continuation phase. Eligible patients were selected randomly from database of each facility, and then were asked for their consent to face-to-face interview.

### Data collection

Structured face-to-face interviews with a paper-based questionnaire were conducted by the 60 trained interviewers who were the employees of the 12 Regional office of Disease Prevention and Control covering the 40 clusters of this survey. One-day training on the interview approach with the survey questionnaire was provided to all interviewers prior to data collection. Questionnaires were adapted to Thai contexts and translated into Thai language (and were pre-tested to ensure the clarity and understandability) from a generic data collection tool provided by the WHO handbook for TB patient cost surveys^11^ comprising four sections: (1) informed consent; (2) patient information (including patient and clinical characteristics, employment, household composition, healthcare utilization, time spent and income lost while seeking and receiving care); (3) costs (i.e., direct medical, direct non-medical, and indirect costs), and time loss before/during the current TB treatment; and (4) coping mechanisms during the treatment phase^15^.

### Data analysis

To estimate direct costs per month, the cost per visit were multiplied by the number of visits per month. The number of visits including outpatient visits, facility-based directly observed therapy (DOT), follow-up, and drug pick-up, of each treatment phase was derived from the national TB control guidelines while direct cost per visit included direct medical cost and direct non-medical cost.

Indirect costs were estimated using a human capital approach. We selected this approach because the proportion of the patients with informal employment in the survey was much higher than other sectors, and this was the better way to present socioeconomic status of the patients based on the Thai context as the consensus from the Thai expert’s consultation. This approach included time lost due to traveling to health facilities and waiting time lost during healthcare consultations of both patients and their household members. The self-reported total time spent on those activities was multiplied by the estimated income per person per minute.

To estimate costs in the remainder of the patient’s current treatment phase (i.e., intensive or continuous phase), extrapolation of the patient's costs in that treatment phase to date was done according to WHO methods^11^. In the case that the costs were estimated for different treatment phases, the mean and median reported costs and number of hours from other patients who were sampled in that treatment phase were used.

Total cost was, then, calculated as the summation of direct medical cost, direct non-medical costs, and indirect costs and was reported for the following treatment stages: pre-diagnosis (from the onset of symptoms to the first visit to a health facility), and post-diagnosis (from first visit to end of treatment).

All cost data were calculated in 2021 value and then converted to USD using the average UN operational rates of exchange during the data collection period (October 2019 to July 2021) of 1 USD = 31.07 THB^16^.

Descriptive statistics were used to describe the participated patients’ characteristics (i.e., genders, age, education level, insurance status, and household size), clinical characteristics (i.e., treatment phase, treatment category, HIV status, type of TB, diagnostic delay, modality of TB treatment, and hospitalization), household economic status (i.e., incomes, expenditures, and impoverishment), costs incurred in TB-affected households, coping strategies, social consequences, social support and perceived financial impact. The proportion of TB-affected household facing catastrophic costs, TB-related total costs (direct and indirect) exceeding 20% of the annual household expenditure as per definition by WHO^11^ and global monitoring^8^ was estimated. Annualized self-reported household expenditure was used as the primary method for determining household ability to pay. In addition, we evaluated pre-disease household poverty levels by comparing daily income (calculated from self-reported household monthly income) against the international poverty threshold of 1.90 USD purchasing power parity^11^ adjusted dollars (converted to PPP by using the PPP conversion factor of 12.34 for Thailand in 2020^17^).

Pearson's chi-square test was applied to compare between patients with first line treatment and patient with drug resistance. Univariate logistic regression analysis was conducted to identify variables associated with facing catastrophic costs due to TB. The variables explored in the univariate analysis included age, sex, employment status, household expenditure quintile, household size, education level, insurance status, HIV status, drug resistance status, TB history, hospitalization during TB episode, mode of TB treatment. Multivariate backward stepwise logistic regression was performed to identify factors affecting catastrophic cost. Adjusted odds ratios (OR) and 95%CI was reported.

### Ethical issues

Prior to the primary data collection of this study, ethical clearance was approved by the Institute for the Development of Human Research Protections (IHRP) (COA No.IHRP2019081 and IHRP No.073-2562), and the Ethical Committee for human research at the Faculty of Dentistry and Faculty of Pharmacy, Mahidol University, Bangkok, Thailand (COA.No.MU-DT/PY-IRB 2018/068.0711 for the initial approval and COA.No.MU-DT/PY-IRB 2020/029.0206 for changes in the sample size). All respondents received a written and oral explanation of the study, and each of them signed an informed consent form before participating in the interview. All methods were performed in accordance with the relevant guidelines and regulations.

## Results

### Patients characteristics

One thousand and four hundred patients (1382 first-line treatment TB and 18 drug-resistant TB, DR-TB patients) in total participated in the costing survey. Table 1 shows the demographic and clinical data for those participants included in the analysis. Most patients were male (68.9%), aged older than 45 years (69.3%) including one quarter over 65 years, had attended pre/primary school education (60.1%), and had public health insurance (98.0%). The median of their household size was three members (range 1–17). The patients who participated in this survey were in any of the two treatment phases with similar proportions (46.1% were in the intensive phase and 53.9% were in the continuation phase). Most patients were new TB (94.4%) without HIV infection (88.2%). Around 31.7% of the patients in the intensive phase experienced a long diagnostic delayed (> 4 weeks). For modality of TB treatment, most patients (75.4%) self-administered their medications, 18.0% of them had home-based directly observed therapy (DOT), and few of them (6.6%) received facility-based DOT. Only 6.6% were hospitalized during their current TB episode, and almost half of them (47.6%) previously hospitalized in their current treatment phase.

**Table 1:** Demographic and clinical characteristics of survey participants.

	TB patients (first-line treatment)		Patients with drug-resistant TB		All TB patients		*p* value
	N	(%)^a^	N	(%)^a^	N	(%)^a^	
Total	1382		18		1400		
Demographic characteristics							
Sex							
Female	428	31.0	7	38.9	435	31.1	0.642
Male	954	69.0	11	61.1	965	68.9	
Age group							
0–14	7	0.5	0	0.0	7	0.5	0.486
15–24	68	4.9	2	11.1	70	5.0	
25–34	142	10.3	4	22.2	146	10.4	
35–44	204	14.8	3	16.7	207	14.8	
45–54	300	21.7	4	22.2	304	21.7	
55–64	299	21.6	2	11.1	301	21.5	
≥ 65	362	26.2	3	16.7	365	26.1	
Education level							
No education	76	5.5	1	5.6	77	5.5	0.920
Pre/primary school	831	60.1	10	55.6	841	60.1	
Secondary school or above	475	34.4	7	38.9	482	34.4	
Employment status (before TB episode)^b^							
Unemployed	217	16.3	3	17.6	220	16.4	0.694
Informal employment	923	69.5	11	64.7	934	69.4	
Formal employment	141	10.6	3	17.6	144	10.7	
Other (retired, student, monk)	47	3.5	0	0.0	47	3.5	
Insurance status							
No insurance	27	2.0	1	5.6	28	2.0	0.812
With insurance	1 355	98.1	17	94.4	1372	98.0	
Household size—median (min–max)	3 (1–17)		4 (1–7)		3 (1–17)		
Clinical characteristics							
Treatment phase							
Intensive phase	638	46.2	7	38.9	645	46.1	0.706
Continuation phase	744	53.8	11	61.1	755	53.9	
Treatment category							
New	1 309	94.7	13	72.2	1 322	94.4	< 0.001
Relapse	60	4.3	3	16.7	63	4.5	
Retreatment	11	0.8	2	11.1	13	0.9	
Unknown	2	0.1	0	0.0	2	0.1	
HIV status							
Negative	1 215	88.2	16	88.9	1231	88.2	0.694
Positive	117	8.5	2	11.1	119	8.5	
Unknown	45	3.3	0	0.0	45	3.2	
Type of TB							
Bacteriologically confirmed pulmonary TB	884	64.0	16	88.9	900	64.3	0.080
Clinically diagnosed pulmonary TB	350	25.3	2	11.1	352	25.2	
Extrapulmonary TB	147	10.6	0	0.0	147	10.5	
Diagnostic delay (> 4 weeks)^c^	224	31.5	5	45.5	229	31.7	0.509
Modality of TB treatment							
Self-administered	1039	75.6	11	61.1	1050	75.4	0.027
Home-based directly observed therapy	247	18.0	3	16.7	250	18.0	
Facility-based directly observed therapy	88	6.4	4	22.2	92	6.6	
Currently hospitalized	90	6.5	2	11.1	92	6.6	0.766
Previously hospitalized in the current treatment phase	647	47.2	14	77.8	661	47.6	0.019

^a^Missing data were excluded from the calculation of proportions described in this table.

^b^Information for employment status from 1345 patients who reported their employment status before TB episode.

^c^Information for diagnostic delay was collected only from patients who were in intensive phase at the time of interview.

### Socio-economic characteristics and the changes in income among TB-affected households

The average monthly income of survey participants and that of their households before the onset of TB symptoms was 355 USD (95%CI 321–388 USD), and 1152 USD (95%CI 708–1597 USD), respectively (Table 2). Almost half of TB patients (48.3%) were the primary income earner. The average monthly household expenditure was 640 USD (95%CI 459–822 USD). While at the interview, the average monthly income of the patient and household decreased to 220 USD (95%CI 193–246 USD), and 643 USD (95%CI 572–714 USD), respectively.

**Table 2:** Self-reported income (2021 USD)^a^ and poverty level.

	TB patients (first-line treatment)		Patients with drug-resistant TB		All TB patients		*p* value
Self-reported monthly Income (in USD)^b^: before onset of TB symptoms, mean^c^ (95% CI)							
Individual patient	354	(320–388)	422	(266–578)	355	(321–388)	0.409
Household	1155	(704–1607)	938	(673–1 202)	1152	(708–1597)	0.462
Self-reported monthly income (in USD)^b^: at the interview, mean^c^ (95% CI)							
Individual patient	222	(195–248)	80	(19–140)	220	(193–246)	< 0.001*
Household	644	(572–716)	565	(332–798)	643	(572–714)	0.517
Monthly expenditure (in USD)^b^, mean (95% CI)							
Household	641	(457–825)	576	(398–754)	640	(459–822)	
	%	(95% CI)	%	(95% CI)	%	(95% CI)	
Patient was the primary income earner before onset of TB symptoms, percentage (95% CI)							
No	51.0	(48.0–54.1)	45.0	(19.6–71.9)	50.9	(47.9–54.0)	0.824
Yes	48.3	(45.3–51.3)	55.0	(28.1–80.4)	48.3	(45.3–51.4)	
Impoverishment: TB-affected households below poverty line^d^, percentage (95% CI) PPP based							
Before onset of TB symptoms	2.3	(1.2–3.7)	0.0	N/A	2.2	(1.2–3.6)	0.583
At the interview	11.1	(8.9–13.6)	11.1	(0.5–32.6)	11.1	(9.0–13.6)	1.000

*CI* confidence interval, *N/A* not applicable, *PPP* purchasing power parity, *TB* tuberculosis, *USD* United States Dollar.

*Significant difference (p < 0.001).

^a^Current value in 2021.

^b^Arithmetic mean.

^c^Income and expenditure were converted to United States Dollars (USD) from Thai Baht (THB) using the average UN Operational Rates of Exchange during data collection period (Apr 2019-Aug 2021) of USD 1 = THB 31.07 (https://treasury.un.org/operationalrates/OperationalRates.php).

^d^Defined as USD 1.90 PPP.

Before the onset of TB symptoms, 2.2% of the participant households faced impoverishment (their incomes were below the poverty line—poverty headcount ratio at USD 1.90 per day at 2011 PPP), and it was increased from 2.2 to 11.1% due to TB (Table 2). The differences in the percentage of impoverishment of TB-affected households before and during TB episodes among the different household income quintile groups are demonstrated in Supplementary (Fig. S1). Our findings show that TB has affected the patients and their households in terms of income loss. The proportion of TB-affected households living below the poverty line was substantially higher among those in lower quintiles.

### Costs of TB-affected households

The mean total costs per TB episode for all study participants (n = 1400) were 903 USD per patient (95%CI 771–1034 USD), and median total costs per episode were 412 USD per patient (IQR 184–879 USD) (Table 3). Of these, total direct non-medical costs were the highest costs (mean, 402 USD, and 95%CI 334–470 USD) incurred per TB-affected households followed by total indirect costs (mean, 393 USD, and 95%CI 315–472 USD) and total direct medical costs (mean, 107 USD, and 95%CI 81–133 USD, respectively. The mean total costs per episode among TB first-line treatment patients (n = 1382) and DR-TB patients (n = 18) were 848 USD (95%CI 725–971 USD) and 4987 USD (95%CI 2884–7090 USD), respectively (Fig. S2 in the Supplementary).

**Table 3:** Detail of costs incurred per TB-affected households (2021 USD)^a^.

TB patient costs^b^, USD	TB patients (first-line treatment)				Patients with drug-resistant TB				All TB patients			
	Mean^c^	(95% CI)	Median	(IQR)	Mean^c^	(95% CI)	Median	(IQR)	Mean^c^	(95% CI)	Median	(IQR)
**Pre-TB diagnosis**												
Direct medical costs	19	(16–23)	14	(1–14)	1	(1–2)	2	(0–2)	19	(16–22)	14	(1–14)
Direct non-medical costs	18	(15–21)	12	(10–12)	14	(8–20)	12	(9–12)	18	(15–21)	12	(10–12)
Total direct costs	37	(33–42)	27	(19–27)	15	(9–22)	14	(10–14)	37	(33–42)	27	(17–27)
Indirect cost	13	(11–14)	7	(2–13)	28	(0–58)	8	(1–16)	13	(11–15)	7	(2–13)
**Post-TB diagnosis**												
Direct medical costs	80	(61–99)	13	(0–76)	485	(228–741)	346	(106–539)	85	(65–105)	19	(0–79)
Direct non-medical costs												
Travel	114	(91–138)	53	(24–111)	657	(332–983)	375	(187–956)	121	(98–145)	54	(24–114)
Accommodation	21	(14–28)	0	(0–13)	80	(28–131)	47	(20–69)	22	(15–29)	0	(0–13)
Food	80	(61–99)	13	(0–76)	485	(228–741)	346	(106–539)	85	(65–105)	19	(0–79)
Nutrition supplement	149	(115–184)	0	(0–120)	622	(0–1403)	–	(0–117)	156	(121–191)	0	(0–120)
Indirect cost	347	(277–416)	92	(24–308)	2927	(1145–4709)	831	(120–4622)	381	(303–458)	94	(25–318)
**Per episode**												
**Total direct medical costs**	**106**	**(80–133)**	**36**	**(14–74)**	**176**	**(106–245)**	**117**	**(21–262)**	**107**	**(81–133)**	**36**	**(14–76)**
**Total direct non-medical costs**	**383**	**(317–448)**	**174**	**(70–391)**	**1857**	**(781–2932)**	**855**	**(526–2390)**	**402**	**(334–470)**	**177**	**(71–404)**
**Total indirect costs**	**359**	**(289–429)**	**106**	**(31–320)**	**2955**	**(1170–4740)**	**835**	**(137–4785)**	**393**	**(315–472)**	**109**	**(31–329)**
**Total cost (human capital approach)**	**848**	**(725–971)**	**406**	**(183–856)**	**4987**	**(2884–7090)**	**2695**	**(1206–8969)**	**903**	**(771–1034)**	**412**	**(184–879)**

*CI* confidence interval, *IQR* interquartile, *TB* tuberculosis, *USD* United States Dollar.

^a^Current value in 2021.

^b^Costs data was converted to United States Dollars (USD) from Thai Baht (THB) using the average UN Operational Rates of Exchange during data collection period (Apr 2019-Aug 2021) of USD1 = THB 31.07 (https://treasury.un.org/operationalrates/OperationalRates.php).

^c^Arithmetic mean.

For the pre-TB diagnosis episode, the mean direct costs (37 USD with 95%CI 33–42 USD) were the highest costs incurred by the patients. The mean total costs incurred during pre-TB diagnosis episode were less than those incurred during post-TB diagnosis episode. Whereas the post-TB diagnosis episode, the mean direct non-medical costs (384 USD with 95%CI 98–191 USD) and the mean indirect costs (381 USD with 95%CI 303–458 USD) were the two highest costs incurred by the patients and their households. This reflects travel, food, and time costs (or productivity lost) by the patients and their caregivers during the TB treatment due to the many facility visits and hour lost (Table 4). In terms of number of facility visits, patients involved in facility-based DOT made 125.8 visits (ranged 114.6–137.0 visits) mainly during their treatment, followed by medical follow-up 9.4 visits (ranged 8.5–10.2 visits). Of these visits, DR-TB patients had significantly higher total number of visits than those of TB patients. Hours lost by DR-TB patients (743.4 h with ranged 350.3–1136.4 h) were also significantly much higher than the lost by TB patients (142.0 h with ranged 122.7–161.3 h). Although hours lost by caregivers were not statistically significant different between TB and DR-TB patients, total lost time of DR-TB caregivers were around four times of those of TB caregivers (372.9 h vs. 85.9 h, respectively).

**Table 4:** Number of facility-visits and hours lost.

	N	TB patients (first-line treatment)		Patients with drug-resistant TB		All TB patients		*p* value
		Number	(95% CI)	Number	(95% CI)	Number	(95% CI)	
Number of facility visits								
Pre-disease	643	2.5	(2.3–2.8)	1.5	(1.1–1.8)	2.5	(2.2–2.8)	< 0.001***
Directly observed therapy	337	119.7	(108.5–130.9)	445.6	(323.3–567.8)	125.8	(114.6–137.0)	< 0.001***
Drug pickup	230	3.7	(2.0–5.4)	10.9	(-1.7–23.6)	3.7	(2.0–5.4)	0.275
Medical follow-up	1400	9.1	(8.2–10.0)	28.5	(18.8–38.3)	9.4	(8.5–10.2)	< 0.001***
Total number of facility visits	1400	39.5	(29.8–49.2)	182.7	(50.8–314.7)	41.4	(31.2–51.6)	0.037*
Hours lost by patient								
Pre-disease	643	7.8	(6.4–9.1)	7.1	(0.8–13.5)	7.8	(6.4–9.2)	0.837
Hospitalization	671	191.4	(159.9–222.8)	706.4	(272.7–1140.1)	202.3	(166.4–238.3)	0.022*
Directly observed therapy	337	23.5	(14.8–32.2)	97.8	(-13.6–209.2)	24.9	(15.5–34.2)	0.192
Drug pickup	230	9.1	(5.7–12.6)	24.5	(-1.6–50.7)	9.3	(5.9–12.7)	0.264
Medical follow-up	1400	38.5	(32.6–44.4)	151.0	(88.1–213.9)	40.0	(34.2–45.8)	0.001**
Total lost time	1400	142.0	(122.7–161.3)	743.4	(350.3–1136.4)	149.9	(127.6–172.3)	0.004**
Hours lost by caregivers								
Hospitalization	671	142.8	(106.7–178.9)	396.6	(-54.8–848.1)	148.2	(107.6–188.9)	0.261
Directly observed therapy	337	3.4	(1.8–5.0)	31.2	(-27.4–89.8)	3.7	(1.6–5.7)	0.351
Drug pickup	230	4.9	(1.9–7.9)	0.0	(0.0–0.0)	4.8	(1.8–7.8)	0.004**
Medical follow-up	1400	41.8	(33.6–50.0)	150.8	(90.6–210.9)	43.2	(35.1–51.4)	0.001**
Total lost time	1400	85.9	(67.9–103.9)	372.9	(55.2–690.6)	89.7	(69.3–110.1)	0.077

*CI* confidence interval, *TB* tuberculosis.

*Significant difference (0.01 ≤ *p* < 0.05).

**Significant difference (0.001 ≤ *p* < 0.01).

***Significant difference (*p* < 0.001).

### Catastrophic total costs

**Figure 1:**
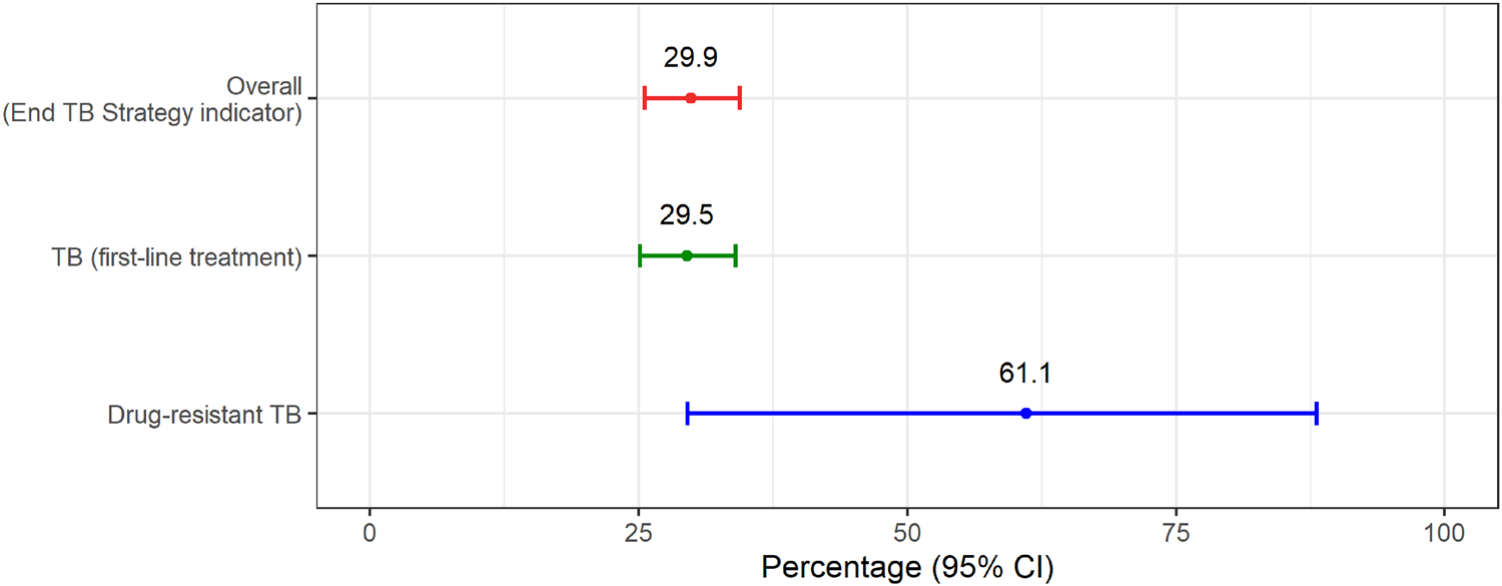
Percentage of TB-affected households facing catastrophic costs. *CI* confidence interval, *TB* tuberculosis. *Error bars represent 95% confidence interval.

Figure 1 illustrates the percentage of TB-affected households facing catastrophic total costs. At the 20% threshold, the percentage of catastrophic total costs was 29.5% (95%CI 25.1–34.0%) for TB and 61.1% (95%CI 29.6–88.1%) for DR-TB patients; this reflects 29.9% of TB-affected households facing catastrophic costs for overall TB participants of this study.

### Coping mechanisms and social consequences

**Figure 2:**
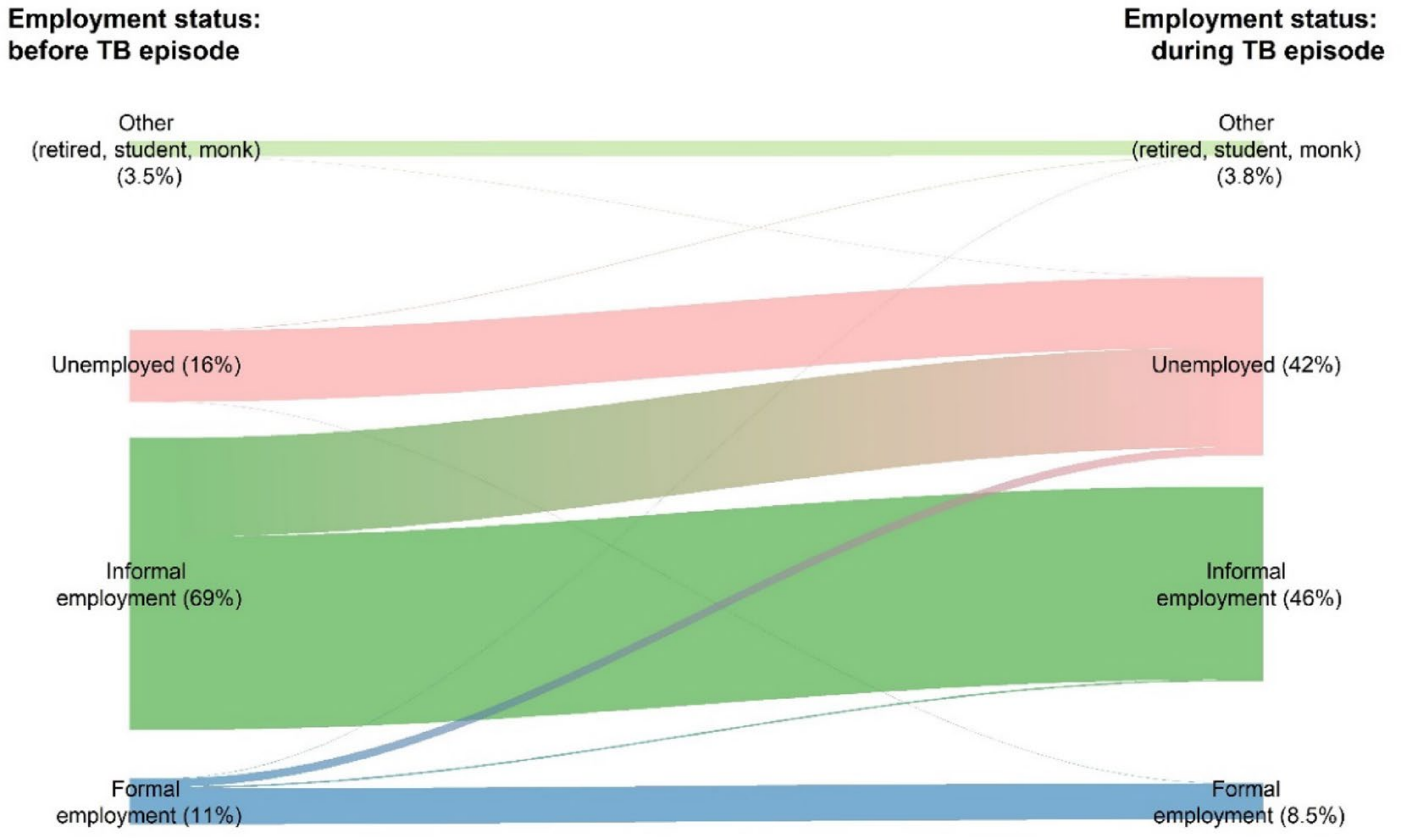
Changes in employment status before and during TB episode.

The patients reported the use of loan as the main coping strategy (19.1%) to face costs incurred with very little social support; 2.2% and 1.0% of survey participants reported receipt of social assistance and vouchers from NTP (Table 5). Getting TB infection causes social consequences, i.e., their working days loss (41.9%), job loss (34.6%), and social exclusion (27.8%). Overall, those proportions of social consequences were significantly higher among DR-TB patients. The proportion of patients who became unemployed more than doubled when comparing the employment status before TB episode to the status during TB episode (at the time of interview) (16.0–42.0%) (Fig. 2). While the proportion of employment in the informal and formal sector decreased from 69.0% and 11.0% to 46.0% and 8.5%, respectively, when comparing the same time periods. More than half of the patients (52.0%) did not perceive any change in the financial impact, while 38.2% of them perceived they were poorer and 8.5% felt they were much poorer than in the past.

**Table 5:** Coping mechanisms and social consequences.

	TB patients (first-line treatment)		Patients with drug-resistant TB		All TB patients		*p* value
	%	(95% CI)	%	(95% CI)	%	(95% CI)	
Coping mechanisms							
Loan	19.1	(15.8–22.6)	22.3	(6.5–44.0)	19.1	(15.9–22.6)	0.715
Sales of assets	5.2	(3.6–7.0)	11.1	(1.2–29.2)	5.3	(3.7–7.2)	0.217
Any of above	21.6	(18.1–25.3)	22.3	(6.5–44.0)	21.6	(18.1–25.3)	0.937
Social consequences							
Food insecurity	4.5	(3.0–6.4)	16.6	(2.7–39.0)	4.7	(3.1–6.5)	0.023*
Divorce/separation	1.8	(1.1–2.8)	5.7	(0.1–24.4)	1.9	(1.1–2.8)	0.254
Job loss	34.3	(30.1–38.5)	55.5	(30.4–79.2)	34.6	(30.6–38.7)	0.079
Interrupted schooling	1.6	(0.9–2.4)	0.0	NA	1.5	(0.9–2.4)	0.606
Social exclusion	27.3	(22.3–32.5)	66.0	(37.9–89.0)	27.8	(22.8–33.1)	0.001**
Working days loss	41.7	(37.3–46.2)	56.1	(32.3–78.4)	41.9	(37.6–46.3)	0.205
Any of above	55.0	(50.1–59.9)	94.2	(75.0–99.9)	55.5	(50.7–60.3)	0.004**
Social support							
Social assistance	2.2	(1.3–3.2)	5.7	(0.1–24.4)	2.2	(1.4–3.3)	0.353
Vouchers from NTP	1.0	(0.2–2.5)	5.7	(0.1–24.4)	1.0	(0.2–2.5)	0.106
Perceived financial impact							
Richer	0.1	(0.0–0.4)	0.0	NA	0.1	(0.0–0.4)	0.087
Not changed	52.4	(47.8–57.0)	21.5	(4.3–46.9)	52.0	(47.5–56.5)	
Poorer	38.0	(34.8–41.2)	55.3	(33.6–76.1)	38.2	(35.1–41.4)	
Much poorer	8.4	(6.0–11.3)	16.8	(3.8–36.4)	8.5	(6.1–11.4)	

*CI* confidence interval, *TB* tuberculosis.

*Significant difference (0.01 ≤ *p* < 0.05).

**Significant difference (0.001 ≤ *p* < 0.01).

### Factors affecting catastrophic costs

**Figure 3:**
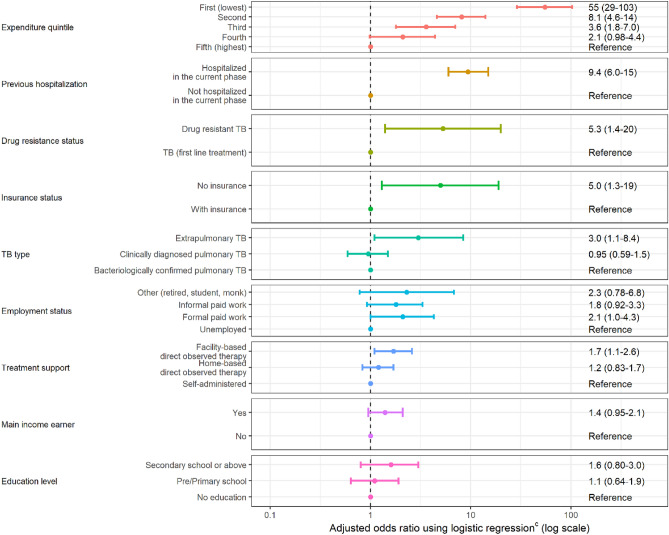
Risk factors for TB-affected households facing costs > 20% of household expenditure due to TB. *Error bars represent 95% confidence interval.

Figure 3 presents the selected final model with adjusted odd ratio (OR) of the risk factors that had a significant association with the probability of facing catastrophic costs due to TB. Households with lower expenditure quintiles (for the first 3 quintiles) had a significantly higher incidence of facing catastrophic costs compared to those in the highest expenditure quintile (the lowest expenditure quintile: OR 54.6, 95%CI 29.0–103.0; the second lowest expenditure quintile: OR 8.1, 95%CI 4.6–14.0, and the third expenditure quintile: OR 3.6, 95%CI 1.8–7.0). The other significant factors associated with the catastrophic costs include experiencing hospitalization (OR 9.4, 95%CI 6.0–15.0, compared to not hospitalizing), being DR-TB patient (OR 5.3, 95%CI 1.4–20.0, compared to those with first-line treatment), patients who do not have health insurance (OR 5.0, 95%CI 1.3–19, compared to those with health insurance), patients with extrapulmonary TB (OR 3.0, 95%CI 1.1–8.4, compared to those with pulmonary TB), and patients who received the facility-based directly observed therapy as their treatment support (OR 1.7, 95%CI 1.1–2.6, compared to those with self-administration).

## Discussion

Our findings illustrated that 29.9% of TB-affected households face catastrophic total costs, a lower proportion compared to the global pooled average of 48% (95%CI 36–61%) with 27 countries with published survey data^8^ and also lower than the global pooled average of 135 low- and middle-income countries with meta-regression estimates 54.9% (47.0–63.2%) overall^9^.

The largest cost driver to the economic burden supported by TB-affected households were travel, food, and nutritional supplementation, in the form of direct non-medical costs (44.5% of total costs), and patient (and their caregivers) productivity loss, in the form of indirect costs (43.6% of total costs).

On the other hand, overall out-of-pocket expenses associated with direct medical costs accounted for only 12% of total costs. Thus, our findings also confirm that most of the direct medical costs have been covered by the Thai public health insurance^18^. Although, almost all direct medical costs during the treatment phase were covered by public health insurance, this study showed that most direct medical costs incurred before TB diagnosis episode were disbursed by patients (pre-diagnosis out-of-pocket expenses represent 2.1% of total episode costs). The patient might seek care by going to the private sector, such as drug store. This could increase out-of-pocket expenses. Thus, increasing proactive access to early TB diagnosis can help early detection of people with TB and bring them to be covered under the public health insurance schemes. Although this has been already included in the Thailand operational plan to end TB for 2017–2021^19^, this finding encourages the Ministry of Public Health to continue this strategy for the next plan to end TB. Moreover, refining benefit packages in all public health insurance schemes to include standard TB care, including diagnosis, treatment and social support is recommended. This can ensure that all presumptive TB cases have access to standard TB treatment.

Although the Thai UHC provides free TB treatment and other medical services, this does not cover traveling and productivity loss incurred from the facility-visits due to TB treatment. Enhancing patient-centered care in the Thai TB treatment guidelines or strengthen all primary health care services may reduce the time required for those facility visits and then decrease the direct non-medical costs and income losses of the patients. Moreover, this has led to another issue of social protection policies that required attention from national policymakers. Social protection policies beyond free medical services, e.g., financial incentives for cost of living, should be strengthened by the national and local government. Only 2.2% (95%CI 1.4–3.3) of survey respondents (Table 5) were accessing social assistance and 1% (95%CI 0.2–2.5) accessed vouchers. For TB patients who are in formal employment, the government should strengthen the policy by securing their jobs. Nevertheless, this issue is not solely the responsibility of government organizations in the health sector, but it also requires cooperation among the health and non-health sectors. Cooperation between The Ministry of Public Health and the Ministry of Labour, the Ministry of Social Development and Human Security, or non-government agencies is required to support TB patients in developing social support mechanism, such as enabling patients to take sick leave or be compensated in case of dismissal, especially for the patients with lower expenditure quintiles. This can mitigate the economic burden and reduce the proportion of households that experience catastrophic costs in Thailand.

Despite the free TB treatment policy under the UHC in Thailand, the percentage of TB-affected households living below the international poverty line^11^ among the TB-affected households increased during TB treatment compared to the pre-TB episode (from 2.2 to 11%). The disease does not affect only to the poor households (percentage living below international poverty line rose from 11 and 0% to 22% and 15% in the 1st and 2nd household income quintiles, respectively) but it also impacts on the richer households (percentage living below international poverty line increased from 0 to 4.7% in the 5th household income quintile). This requires policy actions beyond the strictly medical and into social protection especially for those who are poorer. In addition to the free medical services during TB treatment, income replacement during TB treatment and the post-TB socioeconomic recovery strategies (e.g., maintain their formal employment, looking for a new job, and re-employment) are also key to protect the patients and their households against financial hardship due to TB.

It is also noteworthy that the mean monthly individual incomes reported by TB (first-line treatment) patients is significantly higher than that reported by patients with DR-TB. In fact, the mean total costs incurred by DR-TB cases were almost 6 times of the costs incurred by TB (first-line treatment) patients, even though Thailand has started shorter DR-TB regimen^20^. This highlights the serious socioeconomic impact of DR-TB on their households.

To our knowledge, this is the first national TB patient cost survey in Thailand using the standardized methodology for cross-sectional survey in TB-affected countries developed by WHO^11^. Our findings do not only deliver the significant indicator of catastrophic costs status due to TB in Thailand to achieve the end TB strategies, but we also provide insights that there were gaps in TB policy implementation that needed to improve.

This study has limitations that have led to some concerns. First, we started the survey in 2019 and data collection was ongoing as COVID-19 pandemic hit. This brought an obstacle to the interview process and many of the related health facilities did not allow the interviewers to go to the field. This may cause recall biases due to the delay of the interview appointment. Moreover, the number of health facility visits and income losses may have been interrupted by the pandemic. These might cause under-reported number of the facility visits and the income losses might be resulted from the pandemic. Second, there were missing income data reported from the patients, especially the ones working in informal sector, even though the interviewers tried to ask them to estimate. This might affect the indirect cost estimation. For those missing ones, the estimations of their individual incomes were based on ascribing a proportion of the household annual income to the individual of the reported one. Third, we did not specifically sample for DR-TB, and randomly selected DR-TB in the random clusters; therefore, our findings due to DR-TB cases may not represent the DR-TB patients in Thailand. Although the costs calculation for DR-TB patients were referred to the national standard practice guideline of the DR-TB, its sample size was small and we did not design our data collection of the DR-TB patients for this survey. However, our findings can highlight the higher economic burden of DR-TB than those incurred by TB patients. Thus, we strongly suggest the further study focusing only on DR-TB patients to examine economic burden and catastrophic total costs incurred in DR-TB patients that can be representative of this specific groups of TB patients in Thailand.

## Conclusion

This study is the first national TB patient cost survey in Thailand. Our findings highlight the economic burden on TB patients and their households and of their falling into deeper poverty and greater unemployment. Travel costs, food/nutritional supplementation, and productivity costs drive total TB episode costs in Thailand and a significant proportion of TB-affected households incur in costs > 20% of household expenditure (i.e. catastrophic total costs). Such evidence suggests financial and social protection mechanisms to mitigate the economic burden of the TB-affected households.

### Supplementary Information

Supplementary Information.

## Data Availability

The datasets used and/or analysed during the current study available from the corresponding author on reasonable request and with permission of the Health System Research Institute.
