# Supplementary material for: Catastrophic costs incurred by tuberculosis affected households from Thailand’s first national tuberculosis patient cost survey
Source: Sci Rep. 2024 May 16;14:11205. doi: 10.1038/s41598-024-56594-1 (PMC11099064; doi:10.1038/s41598-024-56594-1)
Supplement: Supplementary file 1 — Supplementary Information. [file 41598_2024_56594_MOESM1_ESM.docx]

**Supplementary**

**Catastrophic costs incurred by tuberculosis-affected households: Thailand’s first national tuberculosis patient cost survey.**

Sitaporn Youngkong^1,2^, Phalin Kamolwat^3^, Phichet Wongrot^2,4^, Montarat Thavorncharoensap^1,2^, Usa Chaikledkaew^1,2^, Sriprapa Nateniyom^3^, Petchawan Pungrassami^3^, Naiyana Praditsitthikorn^5^, Surakameth Mahasirimongkol^6^, Jiraphun Jittikoon^7^, Nobuyuki Nishikiori^8^, Ines Garcia Baena^8^, Takuya Yamanaka^8, 9, 10^

^1^ Mahidol University Health Technology Assessment (MUHTA) Graduate Program, Mahidol University, Thailand

^2^ Social and Administrative Pharmacy Division, Department of Pharmacy, Faculty of Pharmacy, Mahidol University, Thailand

^3^ Division of Tuberculosis, Department of Disease Control, Ministry of Public Health, Thailand

^4^ Faculty of Nursing, Mahidol University, Thailand

^5^ Department of Disease Control, Ministry of Public Health, Thailand

^6^ Department of Medical Sciences, Ministry of Public Health, Thailand

^7^ Department of Biochemistry, Faculty of Pharmacy, Mahidol University, Thailand

^8^ World Health Organization Global Tuberculosis Programme, Geneva, Switzerland

^9^ Department of Global Health and Development, London School of Hygiene & Tropical Medicine, London, UK

^10^ School of Tropical Medicine and Global Health, Nagasaki University, Nagasaki, Japan

Corresponding author:

Sitaporn Youngkong, Ph.D.

Social and Administrative Pharmacy Division, Department of Pharmacy, Faculty of Pharmacy, Mahidol University, 447 Sri-Ayudhaya Rd., Phayathai, Ratchathevi, Bangkok 10400, Thailand

E-mail: sitaporn.you@mahidol.edu


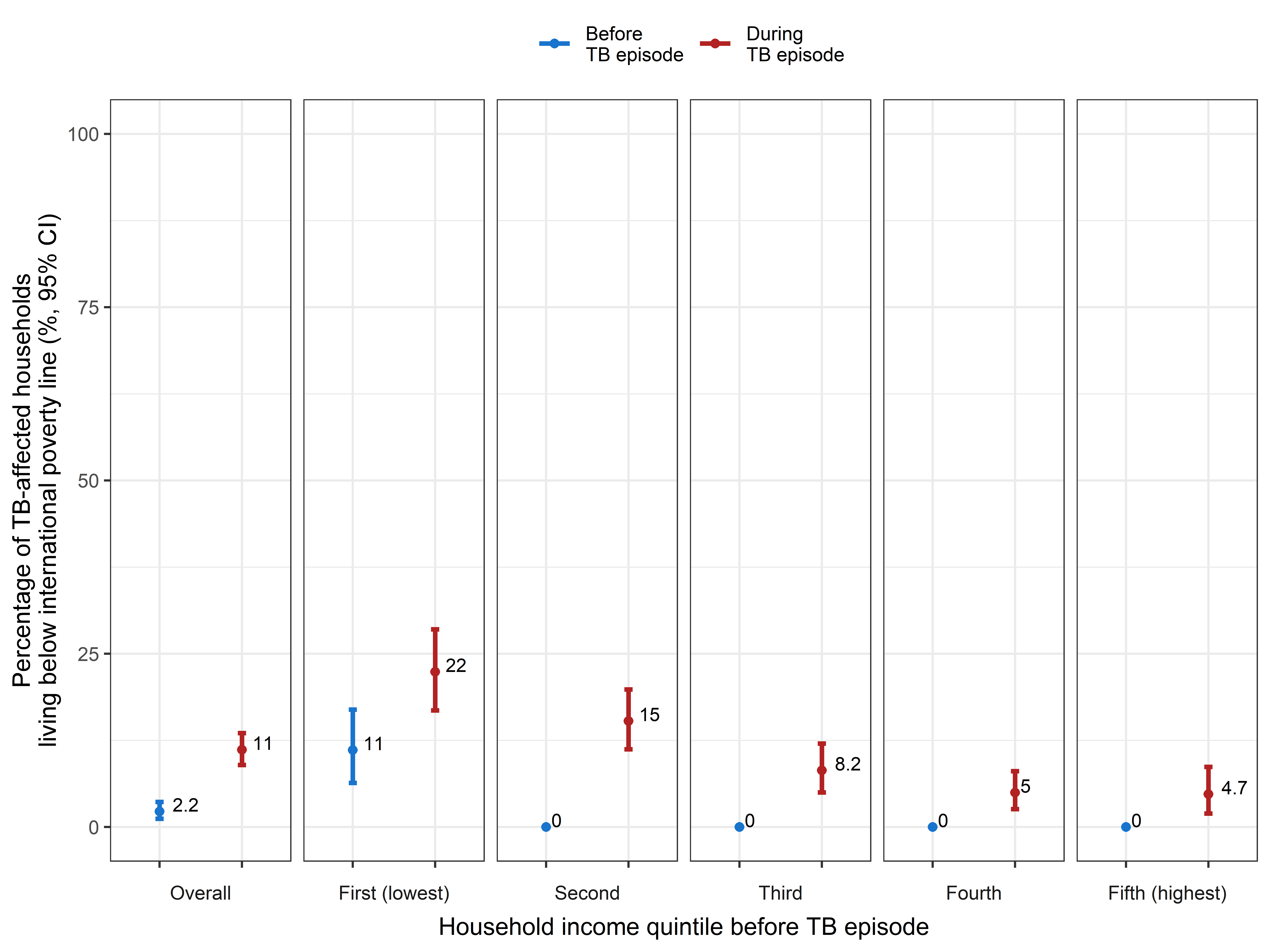


**Figure S1 Impoverishment of TB-affected households during TB treatment (in PPP)**


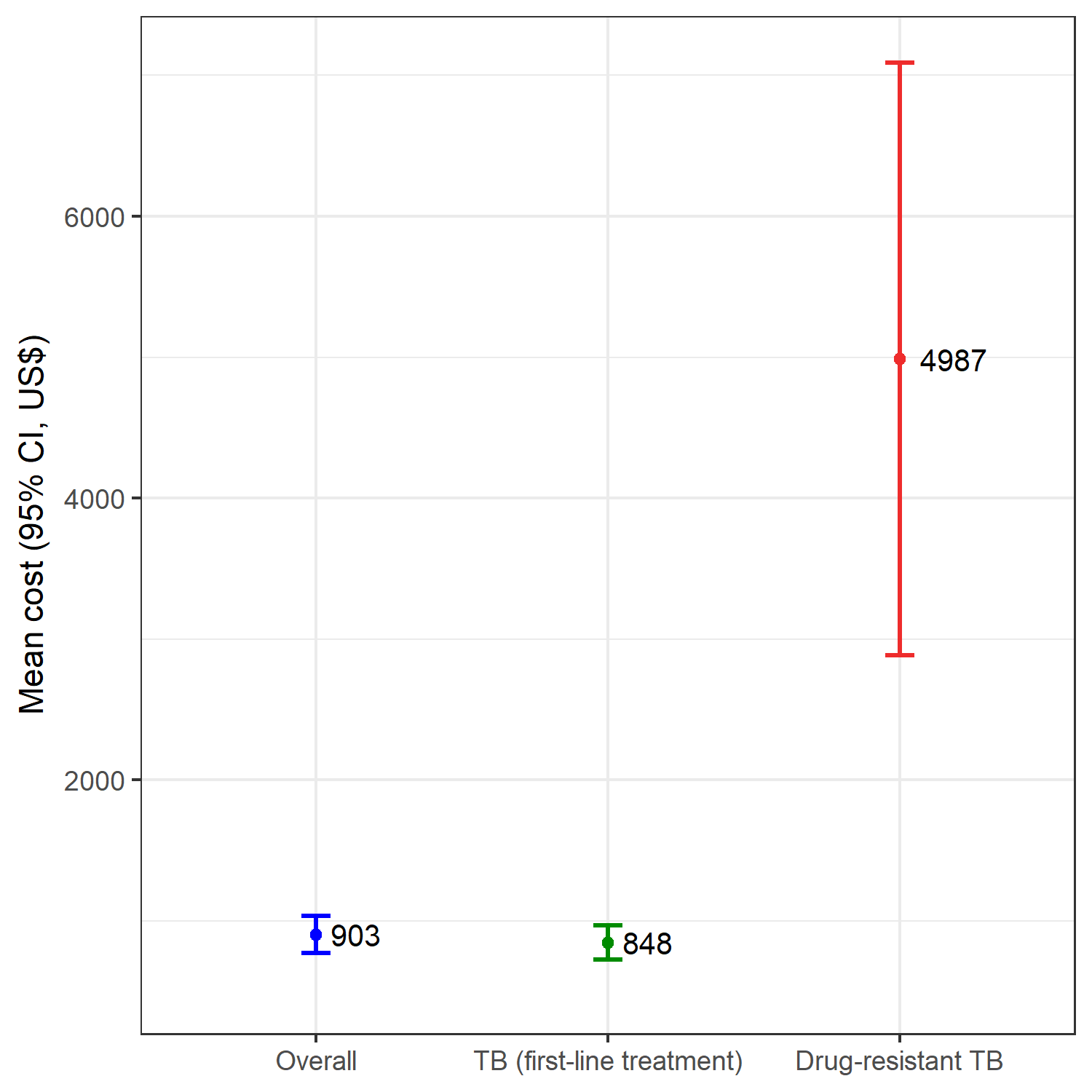


**Figure S2 Total cost incurred per TB-affected household (current 2020 USD)**

**Table S1 Risk factors for catastrophic costs**

| **Risk factors** |  | **Univariate analysis** | | | **Multivariate analysis** | | |
| --- | --- | --- | --- | --- | --- | --- | --- |
|  |  | **Crude OR** | **95% CI** | **p-value** | **Adjusted OR** | **95% CI** | **p-value** |
| **Age group (year)** | 0-14 | 1.4 | (0.3-6.8) | 0.698 | - | - | - |
|  | 15-24 | 0.9 | (0.5-1.7) | 0.781 | - | - | - |
|  | 25-34 | Ref | - | - | - | - | - |
|  | 35-44 | 1.4 | (0.7-2.6) | 0.322 | - | - | - |
|  | 45-54 | 1.5 | (0.9-2.5) | 0.129 | - | - | - |
|  | 55-64 | 1.4 | (0.9-2.2) | 0.128 | - | - | - |
|  | 65+ | 1.8 | (1.1-3.0) | 0.029* | - | - | - |
| **Sex** | Female | Ref | - | - | - | - | - |
|  | Male | 0.9 | (0.7-1.1) | 0.473 | - | - | - |
| **Drug resistance status** | TB (first line treatment) | Ref | - | - | Ref | - | - |
|  | Drug resistant TB | 3.8 | (1.2-12.2) | 0.034* | 10.2 | (3.0-34.8) | 0.001** |
| **Type of TB** | Bacteriologically confirmed pulmonary TB | Ref | - | - | Ref | - | - |
|  | Clinically diagnosed pulmonary TB | 0.8 | (0.6-1.1) | 0.205 | 0.9 | (0.6-1.5) | 0.809 |
|  | Extrapulmonary TB | 1.1 | (0.8-1.6) | 0.461 | 3.0 | (1.2-7.9) | 0.037* |
| **HIV status** | Negative | Ref | - | - | - | - | - |
|  | Positive | 0.9 | (0.7-1.2) | 0.370 | - | - | - |
|  | Unknown | 0.6 | (0.3-1.2) | 0.162 | - | - | - |
| **Treatment group** | New | Ref | - | - | - | - | - |
|  | Relapse | 1.7 | (1.0-2.9) | 0.046* | - | - | - |
|  | Retreatment | 1.0 | (0.4-2.8) | 0.933 | - | - | - |
| **Employment status before TB** | Unemployed | Ref | - | - | Ref | - | - |
|  | Formal employment | 1.0 | (0.7-1.4) | 0.873 | 2.1 | (1.1-4.1) | 0.048* |
|  | Informal employment | 1.3 | (0.9-1.9) | 0.114 | 1.8 | (1.0-3.2) | 0.084 |
|  | Other (student, retired, monk) | 0.9 | (0.4-1.7) | 0.705 | 2.3 | (0.8-6.3) | 0.125 |
| **Education level** | No education | Ref | - | - | Ref | - | - |
|  | Pre/Primary school | 1.2 | (0.8-1.7) | 0.480 | 1.1 | (0.7-1.8) | 0.725 |
|  | Secondary school or above | 1.1 | (0.7-1.7) | 0.723 | 1.6 | (0.8-2.9) | 0.176 |
| **Insurance status** | With insurance | Ref | - | - | Ref | - | - |
|  | No insurance | 1.6 | (0.7-3.6) | 0.311 | 5.0 | (1.4-17.2) | 0.021* |
| **Household size** | ≥3 | Ref | - | - | - | - | - |
|  | <3 | 1.5 | (1.1-1.9) | 0.015* | - | - | - |
| **Household expenditure quintile** | Fifth (highest) | Ref | - | - | Ref | - | - |
|  | Fourth | 1.5 | (0.8-2.8) | 0.237 | 2.1 | (1.0-4.2) | 0.056 |
|  | Third | 2.4 | (1.4-4.1) | 0.003* | 3.6 | (1.9-6.7) | 0.001* |
|  | Second | 5.1 | (3.3-8.0) | <0.001* | 8.1 | (4.8-13.7) | <0.001* |
|  | First (lowest) | 19.1 | (11.4-32.1) | <0.001* | 54.6 | (30.3-98.5) | <0.001* |
| **Treatment support** | Self-administered | Ref | - | - | Ref | - | - |
|  | Home-based directly observed therapy | 1.1 | (0.8-1.5) | 0.626 | 1.2 | (0.9-1.7) | 0.301 |
|  | Facility-based directly observed therapy | 2.9 | (1.7-4.9) | 0.001* | 1.7 | (1.1-2.5) | 0.022* |
| **Primary income earner** | No | Ref | - | - | Ref | - | - |
|  | Yes | 1.5 | (1.2-1.9) | 0.002* | 1.4 | (1.0-2.0) | 0.063 |
| **Current hospitalization** | Currently not hospitalized | Ref | - | - | - | - | - |
|  | Currently hospitalized | 2.7 | (1.6-4.3) | <0.001* | - | - | - |
| **Previous hospitalization** | Previously not hospitalized | Ref | - | - | Ref | - | - |
|  | Previously hospitalized | 4.6 | (3.4-6.2) | <0.001* | 2.2 | (1.7-2.8) | <0.001* |
